# Experimental research of oily sawdust air gasification

**DOI:** 10.1007/s11356-019-06736-0

**Published:** 2019-12-14

**Authors:** Grzegorz Gałko, Danuta Król

**Affiliations:** 1 Institute for Chemical Processing of Coal, Zamkowa 1, 41-803 Zabrze, Poland; 2 Silesian University of Technology, Faculty of Energy and Environmental Engineering, Konarskiego 18, 44-100 Gliwice, Poland

**Keywords:** Gasification, Biomass, Countercurrent reactor, Technological parameter, Methanization coefficient

## Abstract

This article shows oily sawdust gasification research on countercurrent installation. Experimental research was on a laboratory scale. The main purpose of the experiment was combustible gas production with higher CH_4_ concentration. Gas concentrations like CO, CO_2_, CH_4_, H_2_, and C_n_H_m_ determine syngas composition. The technological parameter’s value defines experimental conditions. Value of this was fuel to air ratio. With fuel to air ratio change, syngas composition was a differential phenomenon where it depended on the process parameters like temperature. Additionally, evaluation of methane formation from CO, H_2_, and CO_2_ was done. Methanization coefficients were based on CO and CO_2_ hydrogenation reactions. Component’s activity was in analogs way to syngas components changed.

## Introduction

Energy production is one of the main aspects related to social and economic existence. Sawdust is one of the main wastes formed during wood processing. The presence of them is mostly observed in industrial sectors like wood elements production, furniture, and construction elements like roof truss. They are characterized by low density, high looseness, and varied particle size. Sawdust is formed through the processes like grinding, cutting, sculpturing, and phasing. Plant oil is one of the main products formed in the food industry and agriculture. However, this material might be observed in services like gastronomy. Because of its composition that contains a high amount of combustible substance, an interesting source of energy might be these materials. It means that waste from them besides fossil fuels in thermal processes as energy carrier might be used. Waste like biomass besides fossil fuels in thermal processes as an energy carrier might be used. (Directive of the European Parliament, 2008, Waste Act of 14th December 2012, 2012) Therefore, one of the main directions is biomass waste disposal using thermal processes. In this solution, energy recovery from the process is important. Energy from the process might be recovered for heat, electricity, or both of them in co-generations systems. Conversion of combustible substances is determined in the presence of components included on combustible substances like carbon or hydrogen (Kirubakaran et al. 2009). In minimization trend, waste thermal disposal is included (2008/98/EC). The main directions of biomass utilization using thermal methods are combustion, pyrolysis, and gasification (2008/98/EC). The considerable effect on technological solutions and environmental pollution has elements like chlorine or sulfur which includes combustible substance. Presence of some substances may cause corrosion and deterioration of the process (Lorenz 2005). High moisture content and low concentration of combustible substance additionally may cause adverse phenomena for autothermal combustion. The minimization of this adverse effect might be solved by higher calorific fuel supplementation (Nikodem 2007, Famielec and Famielec 2016). Raw material as waste by thermal transformation besides to energy recovery to post-process ashes might be transformed (Hawrot-Paw et al. 2017). Further, post-process ashes as a substrate for land reclamation and road construction might be widely used. Gasification is one basic method of waste thermal transformation (Dz.U.2013 poz.21). Combustible gas called syngas composed from CO, H2, CH4, CO2, and higher hydrocarbons (CnHm) is the main product of gasification. This gas is the final product of interaction between feedstock and gasification agent. Fuel gas quality is determined by the presence of combustible and incombustible components (Bach-Oller et al. 2015). Concentrations of them depend on the primary and secondary reactions. For example, in some conditions, carbon dioxide might be reduced to carbon monoxide (Emami-Taba and Faisal Irfan 2013, Ledakowicz and Stolarek 2003, Król and Poskrobko 2017). This phenomenon in ranges of temperatures 300 to 900 °C might be observed (Hunt et al. 2013). In installations called gas generators, a gasification process might be conducted. Substrates like fuel and gasification agent as co-current and countercurrent can be delivered. Chamber of the reactors constantly bed, fluidized bed, and circulating as bed included can be used (Puig-Avmat et al. 2010). Directly in combustion engines and energy boilers, syngas as product might be widely used (Sharma et al. 2018). In associated energy systems like boiler and turbine combustible or in chemical synthesis, syngas might be used. Oils and alcohol are some additional products from syngas received (Grzywa and Molenda 1987). In this paper, experimental results of oily sawdust gasification are present.

## Aim of the research

Optimal conditions search for sawdust mixed with oil gasification were the aim of this paper. Countercurrent reactor in the laboratory scale was experimentally researched. Evaluation of methanization substrate reactivity was dependent on coefficients value. Dependency was between fuel to air ratio and syngas composition. The technological parameter value was in agreement with the equation appointed as Eq. (1).

1 $$ \varPhi =\frac{G_{\mathrm{fuel}}}{G_{\mathrm{air}}},- $$

where *G*_fuel_ is fuel stream delivered (kg·h^−1^) and *G*_air_ is air stream delivered (kg·h^−1^).

Research was with constant stream of gasification agent and diversified stream of fuel. Parameter values are in Table 1.

**Table 1:** Fuel and air stream in dependence of technological parameter value

Technological parameter *Φ*, −	Fuel stream, *G*_fuel_ kg·h^−^1	Air stream, *G*_air_ kg·h^−^1
0.32	0.151	0.472
1.19	0.562	
2.06	0.972	

A stable stream of gasification agent with 0.472 kg/h−1value was delivered to the chamber. Fuel delivery to the chamber was in the range from 0.151 to 0.972 kg/h−1. Evaluation of methane formation, gas activity was determined.

## Gasification process

Gasification is a reaction cycle of the between fuel and gasification agent. The main product of the process is combustible syngas. Process intensity depends on pressure and temperature (Emami-Taba and Faisal Irfan 2013; Bach-Oller et al. 2015). Gasification reactions belong to the endothermal and exothermal groups (Tomeczek 1991). Example reactions that participate in the process are the following:

- Steam carbon monoxide conversion:
  2 $$ \mathrm{CO}+{\mathrm{H}}_2\mathrm{O}\to {\mathrm{CO}}_2+{\mathrm{H}}_2 $$
- Steam reforming:
  3 $$ {\mathrm{CH}}_4+{\mathrm{H}}_2\mathrm{O}\to \mathrm{CO}+3{\mathrm{H}}_2 $$
- Methanization:(carbon monoxide hydrogenation):
  4 $$ \mathrm{CO}+3{\mathrm{H}}_2\to {\mathrm{CH}}_4+{\mathrm{H}}_2\mathrm{O} $$
- Methanization (carbon dioxide hydrogenation)
  5 $$ {\mathrm{CO}}_2+3{\mathrm{H}}_2\to {\mathrm{CH}}_4+{\mathrm{H}}_2\mathrm{O} $$
- Dry reforming
  6 $$ {\mathrm{CH}}_4+{\mathrm{CO}}_2\to 2\mathrm{CO}+2{\mathrm{H}}_2 $$

Syngas calorific value is in correlation with concentrations of combustible gases (Bach-Oller et al. 2015, Król and Gałko 2017). The composition of gases depends on the type and chemical reactions intensity (Bach-Oller et al. 2015; Emami-Taba and Faisal Irfan 2013).

## Materials and methods

In the form of post-production waste, coniferous sawdust for research was selected. This material is usually generated in processes related to wood processing. Sawdust with plant oil made from sunflowers was blended in mass ratio 1:1. Fuel analysis by actual standards was done. Measurements are shown in Table 2.

**Table 2:** Fuel properties

Parameter	Value
C^d^, %	56.31
H^d^, %	6.75
N^d^, %	0.85
S^d^, %	0.13
Cl^d^, %	0.33
O^d^, %	20.61
A^d^, %	15.02
Combustible substance^d^, %	84.98
Moisture^r^, %	6.00
Lower heating value^d^, MJ/kg^−1^	23.626

Explanations (condition of fuel): ^d^dry basis; ^r^as received (with moisture)

The content of carbon included in the combustible substance was 56.31%. Ash content in fuel was 15.02%. Content of carbon in comparison with hard coals was lower. Usually, carbon content in coal is around 80%. Ash and moisture to fuel part called ballast belongs. Calorific value decreases by the presence of them is caused.

### Research placement

**Fig. 1:**
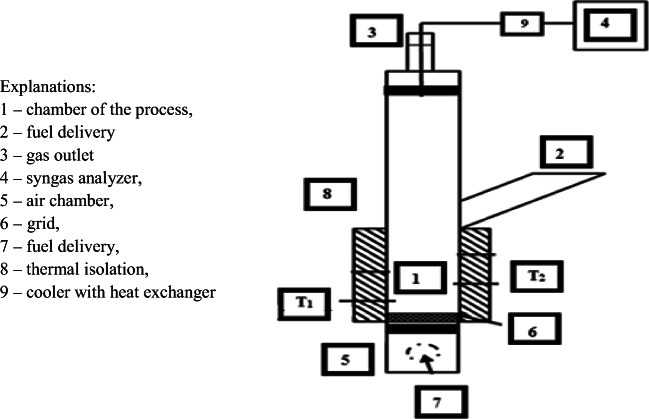
Research placement. (1) Chamber of the process, (2) fuel delivery, (3) gas outlet, (4) syngas analyzer, (5) air chamber, (6) grid, (7) gasification agent delivery, (8) thermal isolation, and (9) cooler with heat exchanger

Experiment on countercurrent installation in laboratory scale was done. Installation from 3 segments with a flange connection was constructed. In the 1st segment, there is a gas outlet with an installed pipe connected syngas analyzer. The 2nd segment was chamber of the process where feedstock delivered to the installation is gasified. The second segment is separated from the 3rd segment by the grid. The chamber of the process is additionally isolated with scheme is shown in Fig 1.

Installation was with usage of fiberglass wool for boiler technology isolated. Air with temperature 25 °C was a gasification agent. Temperatures were over the grid and fuel layer using nickel thermocouples “type K” measured. Combustible gas composition using analyzer type GAS 3100 syngas analyzer, including CO, CO_2_, CH_4_, H_2_, and C_n_H_m_ was measured. Additionally, before analysis, gas using a thermoelectric cooler type “EZ Clean” heat exchanger was cooled. After cooling with countercurrently connected filter systems, syngas was cleaned.

### Methanization coefficients evaluation

As part of the research evaluation of substrates, activity on methane formation using coefficients was done. Substrate concentrations are based on methanization activity coefficients value (Frusteri et al. 2017). Some groups of methanization substrates are products that originate from primary reactions between fuel and gasification agent (Frusteri et al. 2017; Król and Poskrobko 2016). One kind of methane formation reaction is Sabatier-Sanders’s carbon dioxide hydrogenation shown in Eq. (7):

7 $$ {\mathrm{CO}}_2+4{\mathrm{H}}_2\Longleftrightarrow {\mathrm{CH}}_4+2{\mathrm{H}}_2\mathrm{O} $$

Carbon monoxide hydrogenation in accordance with Fischer-Tropsch is the second kind of reaction:

8 $$ \mathrm{CO}+3{\mathrm{H}}_2\to {\mathrm{CH}}_4+{\mathrm{H}}_2\mathrm{O} $$

The methanization substrates activity was calculated with Eq. (9).

9 $$ {W}_{\mathrm{mx}}=\frac{{\mathrm{CH}}_4}{\mathrm{zX}},-\kern1em $$

where *W*_mx_ is methanization coefficient (−), zX is methanization substrate %, and CH_4_ is methane in syngas concentration (%).

## Results

Ranges of average temperatures from the experiment are shown in Table 3. Temperatures over the grid and over the fuel layer were measured.

**Table 3:** Average temperatures of process

Temperature over the grid, °C	Temperature over the fuel layer, °C
475–500	790–820

**Fig. 2:**
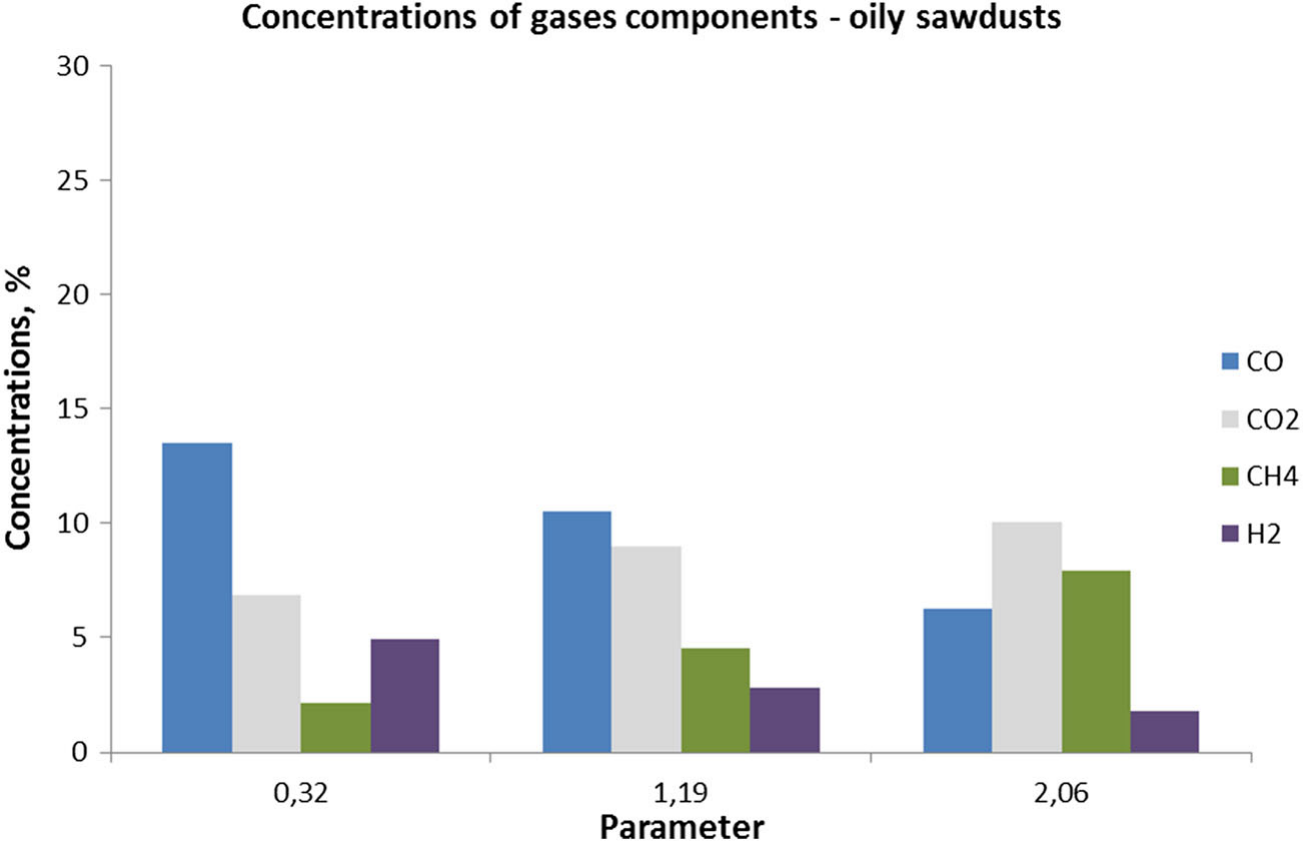
Gaseous concentrations in function of technological parameter (Φ) value

With separation for over the grid and fuel, layer temperature distribution was visible. Over the fuel layer than over the grid in higher ranges temperatures were observed. Biomass gasification results in the function of fuel to air ratio are shown in Fig. 2.

In dependence from fuel to air ratio changes, syngas calorific values are shown in Table 4.

**Table 4:** Syngas LHV in dependence from technological parameter value

Technological parameter Φ, −	LHV, MJ·(m3n)−1
0.32	2.84
1.19	3.24
2.06	3.86

**Fig. 3:**
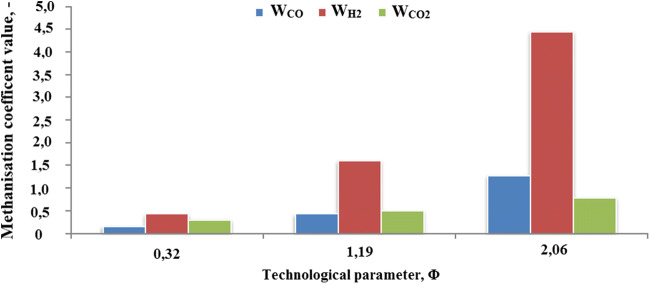
Changes of methanization coefficients values in function of fuel to air ratio increase *Φ*

It was found that combustible syngas formation from gasification of oily sawdust as a feedstock is possible. The opportunity is possible because of the physical and chemical properties of this material. Its contents were in the range from 7 to 14%. The greatest concentration for carbon monoxide was observed. Increased concentration in the range of 2 to 8% for methane was noticed. Usually, CH_4_ concentration is in the range from 3 to 4%. With fuel to air ratio grew up, methane concentrations were increased. In correlation with carbon monoxide and hydrogen decrease, this phenomenon was observed. If the temperature is lower than for cracking conditions, methane concentration increase is possible. Usually, if the temperature is over 800 °C, this phenomenon happens (Lan et al. 2019). Additionally, higher concentrations with the range 7 to 10% for incombustible CO_2_ were observed. Evaluation of energy potential led to the conclusion that syngas calorific value with fuel to air ratio grew up was increased. As a result, the greatest influence on combustible syngas calorific value grew up with CH_4_ concentration. Six times fuel to air ratio increase has caused a 30% increase of calorific value. Substrate type methanization coefficients were determined. Changes of methanization coefficients in correlation from fuel to air ratio are shown in Fig. 3.

Process temperatures were in the 475–500 °C range on the grid and 790–820 °C on the fuel layer. As the ratio of fuel to air increased, H_2_ and CO concentrations were reduced. On the other hand, an increase in CH_4_ and H_2_ was observed. Concentrations and methanization rates increased with the CO and H_2_ reduction. A slight decrease in the CO coefficient was observed. It follows that in methane production, the most active substrates were hydrogen and carbon dioxide. This indicates favorable conditions for Fischer-Tropsch and Sabatier-Sander reactions.

## Conclusions

Based on carried experimental research, it was found that an alternative for other oily sawdust thermal transformation for processes like pyrolysis and combustion is a gasification process. Syngas composition with fuel to air ratio increase was changed. At the same time, substrate content changes affect its amount and quality products received. The mass and energy balance of the process with components concentrations was changed. By the lowest fuel to air ratio and increase of methane concentration in correlation with fuel to air ratio increase, the highest carbon monoxide concentration was observed. By methanization coefficients, value changes of activity components were depended. Syngas with CH_4_ higher concentration in small cubature furnaces might be widely used. High methane concentration improves the conditions favorable for the laminar flame stability. It also might be widely used in small dispersed energy systems for heat, electricity, or both of them in co-generation systems. The main purpose of dispersed energy systems is using energy recovery near the place of production from easily available materials. Due to energy cost production increase, combustible syngas from oily sawdust gasification might be an interesting solution in “off-grid” autonomous systems. If energy systems’ modernization, construction, or magnification is not justified, such practices are usually observed. For such a solution, an additional argument is the huge availability of raw materials. Side products of wood processing do not require special transformation. An effective solution is oily sawdust gasification. It solves a waste management problem with heat energy recovery. With actual regulations, there fits a trend of a friendly environmental economy with principles of sustainable development including all of these activities.
